# Circulating inflammatory biomarkers and risk of intracranial aneurysm: a Mendelian randomization study

**DOI:** 10.1186/s40001-023-01609-2

**Published:** 2024-01-03

**Authors:** Jianxun Fang, Yuze Cao, Jun Ni

**Affiliations:** Department of Neurology, State Key Laboratory of Complex Severe and Rare Diseases, Peking Union Medical College Hospital, Chinese Academy of Medical Sciences and Peking Union Medical College, No 1, Shuaifuyuan, Dongcheng District, Beijing, 100730 China

**Keywords:** Cytokines, Inflammation, Intracranial aneurysm, Mendelian randomization

## Abstract

**Background:**

Intracranial aneurysm (IA) accounts for a substantial source of non-traumatic subarachnoid hemorrhage, with inflammation postulated as a potential factor in its pathogenesis. The present study aims at evaluating the association between circulating inflammatory cytokines and risk of IA under a bidirectional two-sample Mendelian randomization (MR) design.

**Methods:**

For primary analysis, summary statistics of inflammatory regulators was obtained from a genome-wide association study (GWAS) comprising 8293 Finnish participants. Summary data of IA were extracted from a GWAS which comprised 7495 cases and 71,934 controls in European descent. For targeted analysis, summary statistics were extracted from two proteomic studies, which recruit 3301 and 5368 European participants, respectively. Summary data of IA were acquired from FinnGen study with 5342 cases and 342,673 controls. We employed inverse variance weighted (IVW) method as main approach, with sensitivity analyses using weighted median, MR-Egger, and MR-PRESSO methods. Reverse MR analyses were conducted to minimize bias from reverse causality.

**Results:**

No causation of cytokines with IA subtypes was identified in both primary and targeted analysis after Bonferroni correction. In primary analysis, vascular endothelial growth factor (VEGF) and fibroblast growth factor basic (bFGF) levels were suggestively associated with aneurysmal subarachnoid hemorrhage (aSAH) [VEGF → aSAH: OR = 1.15, 95%CI  1.04–1.26, *P* = 0.005; bFGF → aSAH: OR = 0.62, 95% CI 0.42–0.92, *P* = 0.02]. Statistical significance failed to replicate in targeted analysis. Instead, suggestive protective effects for aSAH were identified in FGF-9 (FGF-9 → aSAH: OR = 0.74, 95% CI 0.62–0.89, *P* = 0.001) and FGF-16 (FGF-16 → aSAH: OR = 0.84, 95% CI 0.72–0.97, *P* = 0.017). Furthermore, reverse analyses identified suggestive effect of unruptured IA on RANTES, MIF, GRO-alpha, FGF-16, and FGF-19. Result remained robust after applying sensitivity tests.

**Conclusions:**

No causality of inflammatory biomarkers on the risk of IA subtypes was identified. Future large-scale studies are in need to evaluate the temporal dynamics of cytokines in conjunction with IA.

**Supplementary Information:**

The online version contains supplementary material available at 10.1186/s40001-023-01609-2.

## Background

Intracranial aneurysm (IA) is a common cerebrovascular lesion characterized by pathological dilation of intracranial arteries. The overall prevalence rate of unruptured intracranial aneurysm (uIA) was estimated to be 3.2% across population without comorbidities, and aneurysmal rupture accounted for 80–85% of non-traumatic subarachnoid hemorrhage (SAH) [1]. Several risk factors were identified from observational studies which revealed hypertension, smoking, old age (> 50 years), female sex, and genetic factors might increase the occurrence of IA, while heavy alcohol use, high dose of estrogen, cocaine use, and low body mass index might increase the risk of aneurysmal subarachnoid hemorrhage (aSAH). However, the causal effect of risk factors to IA remained elusive [2].

Precise molecular mechanisms of IA formation, growth, and rupture are incompletely elucidated. Still, accumulating evidence has suggested potential role of inflammation in the pathogenesis of IA. Cellular and humoral inflammatory responses may promote influx of macrophages, degradation of extracellular matrix, and disruption the stability of arterial wall [3]. The regulation of this particular process has been determined to be mediated by circulating proinflammatory cytokines including tumor necrosis factor (TNF), monocyte chemoattractant protein‑1 (MCP-1), interleukin-1 betta (IL‑1β), and matrix metalloproteinases (MMP) [4, 5]. Furthermore, the key inflammatory enzymes cyclooxygenase‑2 (COX‑2) was hypothesized to be associated with aneurysm stability as COX-2 was more abundantly observed in arterial wall of ruptured aneurysm [6].

Mendelian randomization (MR) is an epidemiologic method which utilizes genetic variants, for instance, single nucleotide polymorphisms (SNPs), as instrumental variables (IVs) to infer the possible causal relationship between exposure and outcome. Due to random allocation of inherited variants during gamete formation, MR analysis may be capable of avoiding the bias of potential confounding effects and reverse causality [7]. Recent studies have utilized MR to explore the casual relationship between IA and various risk factors, with only one study examining potential inflammatory mechanism. With a hypothesis-driven design, previous MR study have revealed sIL6R and C-reactive protein (CRP) levels were not associated with IA risk [8]. Given these uncertainties, we designed this MR study to further assesses whether causality exists between inflammatory biomarkers and IA.

In the present study, by leveraging genetic data from genome-wide association study (GWAS) and publicly accessible dataset, we conducted the first bidirectional two-sample MR analysis to systematically screen for the possible causality between 41 circulating inflammatory biomarkers and the risk of aSAH and uIA.

## Methods

### Study design

**Fig. 1:**
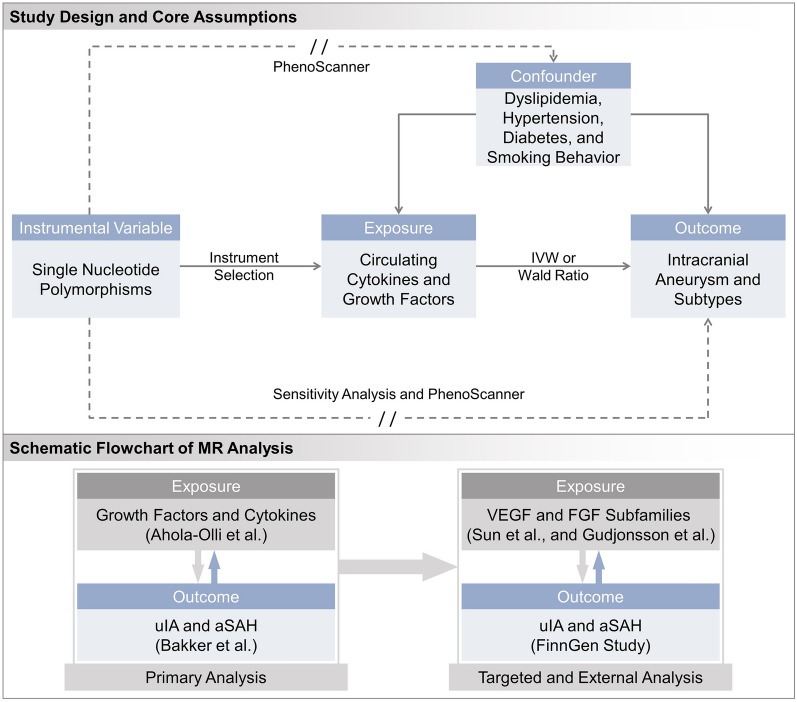
Overall design for Mendelian randomization study. *MR* Mendelian randomization, *IVW* inverse variance weighted method, *IA* intracranial aneurysm, *aSAH* aneurysmal subarachnoid hemorrhage, *uIA* unruptured intracranial aneurysm, *VEGF* vascular endothelial growth factor, *FGF* fibroblastic growth factor

This is a bidirectional two-sample Mendelian randomization study aiming at investigating the association of inflammatory biomarkers and IA, utilizing SNPs as genetic instrument. The overview of study design is illustrated in Fig. 1. The accuracy and efficacy of MR analysis relied on three assumptions: (1) the IVs should be strongly related to exposure; (2) the IVs should not be associated with confounders which may influence exposure and outcome; (3) the IVs could only influence outcome through the exposure [9]. In the present study, the first assumption was evaluated through the selection process of IVs. The second and third assumptions, could not be empirically proven and were assessed through sensitivity analyses and systemically pleiotropic scanning by the investigators.

### Data sources

Aggregate data used in the present study are listed in Additional file 1: Table S1. In primary analysis, summary statistics of inflammatory regulators were obtained from publicly available GWAS, which included two research cohorts (*The Cardiovascular Risk in Young Finns Study* and *FINRISK*) comprising 8293 Finnish participants in and investigated the genetic associations of 41 circulating inflammatory cytokines and growth factors [10]. Summary-level data of intracranial aneurysm and its subtype were extracted from a GWAS conducted by The International Stroke Genetics Consortium (ISGC) Intracranial Aneurysm Working Group, which comprised approximately 79,429 participants in European descent (7495 cases and 71,934 controls for IA; 2070 cases vs. 71,952 controls for uIA; 5140 cases and 71,952 controls for aSAH). Data were accessed through the ISGC Cerebrovascular Disease Knowledge Portal [11].

For targeted and external analysis, in order to construct a comprehensive plasma spectrum of vascular endothelial growth factor (VEGF) and fibroblast growth factor (FGF) families, summary statistics were extracted from two distinct study groups. The first was a proteomic study which investigated 3622 plasma proteins in 3301 healthy European participants from the INTERVAL cohort [12], and the second proteogenomic study analyzed 2091 serum proteins in 5368 individuals with European ancestry [13]. Summary data of IA were acquired from FinnGen study, which aimed at collecting and analyzing genome and health data from 500,000 Finnish biobank participants. Specifically, it comprised 2548 cases of uIA, 5342 cases of aSAH and postoperative IA, and 342,673 controls. Data of overall IA statistics were not available in FinnGen study [14].

### Instrument selection

Genetic instrumental variables were defined as SNPs significantly and independently associated with 41 cytokines and growth factors in primary analysis. Based upon preliminary calculation, it was observed that only 11 out of 41 cytokines had more than 3 SNPs after removal of duplicated variables and clumping, as determined by a cut-off value of *P* < 5 × 10^-8^ (Additional file 1: Table S2). It was not sufficient for further pleiotropic analysis, therefore, a more relaxed threshold at *P* < 5 × 10^-6^ was used in the present study [15]. To avoid bias from linkage disequilibrium within SNPs, instrumental variables were clumped with exclusion criterion of *r*^2^ > 0.1 facilitated by 1000 Genome Project using European ancestry. Duplicated SNPs related to multiple biomarkers were excluded. Proxy SNPs were acquired from LDlink (https://ldlink.nih.gov/) with selection criterion of R^2^ > 0.8 when SNPs were not available in the outcome data [16]. Variation explained by each genetic instrument was determined by the formula: 2 × EAF × (1—EAF) × beta^2^, where EAF is the effect allele frequency and beta is the estimated effect. Total variance explained (R^2^) for each exposure was computed as the cumulative value for each SNP [17]. *F*-statistics (*F* = beta^2^/se^2^, where beta is the estimated effect and se is the standard error) was used to assess the strength of individual IV, and instruments with *F* < 10 were considered weak and excluded. Total strength of each exposure was represented by the mean value of *F*-statistics [18, 19]. The same selection criterion was applied to targeted analysis, with VEGF and FGF subtypes being exposures. For reverse analysis, SNPs were extracted from the initial outcome dataset in forward analysis and underwent the identical selection process.

### Mendelian randomization analysis

Our study involved the following analytic processes conducted through bidirectional MR: (1) effect of inflammatory biomarkers on IA, and corresponding reverse analysis; (2) effect of VEGF and FGF subtypes on IA, and corresponding reverse analysis. Aggregate statistics of exposure and outcome were assembled and harmonized. Palindromic SNPs which failed to infer proper effect allele frequency were excluded [20]. In the main analysis, two methodologies were employed: the Wald ratio and the inverse variance weighted (IVW) techniques. Specifically, the Wald ratio was calculated for exposures with only one SNP, while IVW was utilized in cases where the exposure was associated with more than one SNP [21]. Additionally, in situations where the SNPs exhibited substantial heterogeneity, the IVW method with multiplicative random effects was used, otherwise IVW with fixed effects was applied [22]. Cochran’s Q statistic was calculated to evaluate the heterogeneity and provide evidence for invalid instruments (*P* < 0.05) [23]. Concerning about the statistical efficacy derived from sample size, statistical power was computed through mRnd (https://shiny.cnsgenomics.com/mRnd/), given 5% as type-1 error rate [24].

### Sensitivity assessment

MR-Steiger test was conducted to assessed directionality, which operated under the assumption that a reliable IV explained more variance in exposure than outcome [25]. Consistency between reverse MR and MR-Steiger results were evaluated. Sensitivity analyses were conducted utilizing weight median, MR-Egger, and MR-PRESSO (Mendelian randomization pleiotropy residual sum and outlier) methods. The weighted median method estimates casual effect assuming at least 50% of the IVs are valid. The MR-Egger analysis estimates causality when InSIDE (the Instrument Strength Independent of Direct Effect) assumption holds, even in the absence of valid IVs. Significance intercept generated by MR-Egger regression indicates pleiotropy (*P* < 0.05) [26]. Both MR-Egger and weighted median methods required at least 3 SNPs for analysis. The MR-PRESSO method tends to identify and remove the SNP outliers with horizontal pleiotropy. Significant results of global test via MR-PRESSO indicate the presence of and horizontal pleiotropy [27], utilizing at least 4 SNPs. Besides, the original instrumental variables set was screened through the QTLbase (http://www.mulinlab.org/qtlbase) and sensitivity analyses utilizing *cis*-association were conducted to further assess the horizontal pleiotropy [28]. Furthermore, for exposures with nominal significance (*P* < 0.05) in the MR analysis, systematic screening for potential phenotypes correlated with confounders (dyslipidemia, hypertension, diabetes, and smoking) was conducted through PhenoScanner (http://www.phenoscanner.medschl.cam.ac.uk/) [29, 30].

### Statistical analysis

All analyses were conducted in R statistical software (version 4.3.0) with R packages “TwoSampleMR” and “MRPRESSO”. For binary outcome, causal effect was expressed as odds ratio (OR) per standard deviation (SD) higher of biomarker levels, accompanied by 95% confidence interval (CI). For continuous outcome, result was expressed as regression coefficient beta (β) and corresponding 95% CI. After Bonferroni correction for multiple comparisons, statistical significance was defined as *P* < 6.2 × 10^-4^ (0.05/80) for primary analysis group, and *P* < 8.6 × 10^-4^ (0.05/58) for external group, respectively. A more expanded cut-off value of *P* < 0.05 was considered nominal or suggestive significance. All statistical tests were two-sided (Additional file 2).

## Results

### Circulating inflammatory biomarkers and risk of IA

In primary analysis, 80 SNPs representing 23 cytokines were selected at P < 5 × 10^-8^, among which, 11 out of 41 cytokines had more than 3 SNPs. It was not sufficient for MR-PRESSO analysis to assess horizontal pleiotropy; therefore, we used a more relaxed threshold. 663 SNPs were identified at P < 5 × 10^-6^. After excluding unmatched SNPs and finding proxies in outcome data, 307 SNPs accounting for 40 cytokines were employed as instrumental variables. Variation explained by individual exposures ranged from 0.81% for IL-6 to 29.6% for MIP-1b. *F*-statistics ranged from 11.71 to 784.0, indicating no potential bias derived from weak instrument (Additional file 1: Table S2).

**Fig. 2:**
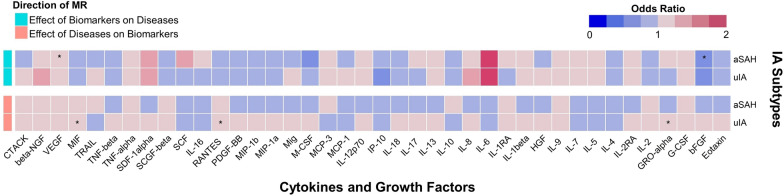
Mendelian randomization associations of inflammatory biomarker levels with intracranial aneurysm. Results were normalized applying the equation: beta = ln (odds ratio). Asterisk (*) indicated nominal significance of *P* < 0.05; *MR* Mendelian randomization, *IA* intracranial aneurysm, *aSAH* aneurysmal subarachnoid hemorrhage, *uIA* unruptured intracranial aneurysm, *CTACK* cutaneous T cell-attracting chemokine, *NGF* nerve growth factor, *VEGF* vascular endothelial growth factor, *MIF* macrophage migration inhibitory factor, *TRAIL* TNF-related apoptosis-inducing ligand, *TNF* tumor necrosis factor, *SDF* Stromal-derived factor, *SCGF* stem cell growth factor, *SCF* stem cell factor, *IL* interleukin, *RANTES* C–C chemokine ligand 5, *PDGF-BB* platelet-derived growth factor-BB, *MIP* macrophage inflammatory protein, *Mig* monokine induced by interferon-gamma, *M-CSF* monocyte colony-stimulating factor, *MCP* monocyte chemoattractant protein, *IP* interferon-gamma-inducible protein, *HGF* hepatocyte growth factor, *GRO-alpha* growth-regulated protein alpha, *G-CSF* granulocyte colony-stimulating factor, *bFGF* fibroblast growth factor basic

**Fig. 3:**
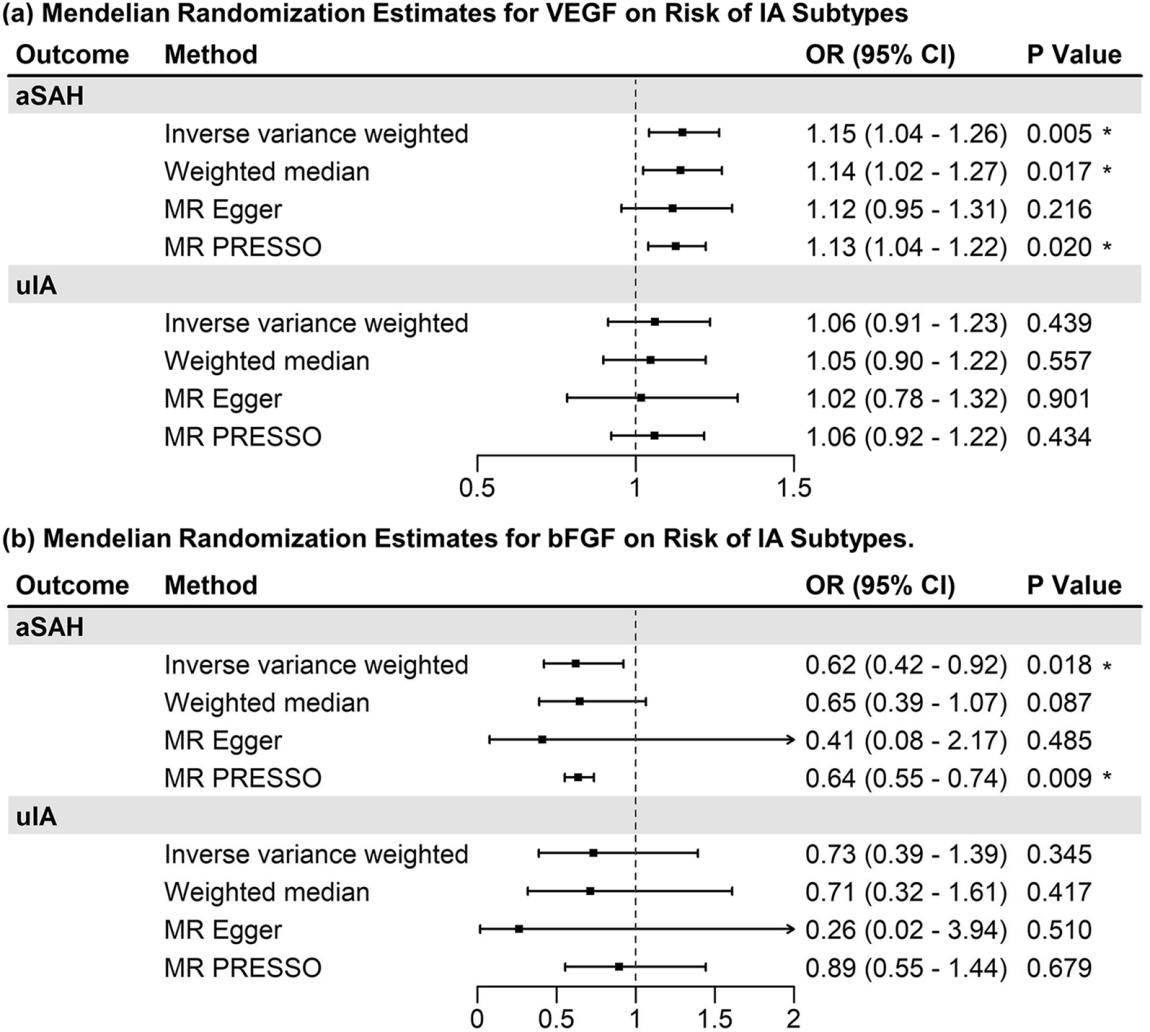
Mendelian randomization estimates utilizing VEGF and bFGF as exposures. Asterisk (*) indicated nominal significance of *P* < 0.05; IA, intracranial aneurysm; *aSAH* aneurysmal subarachnoid hemorrhage, *uIA* unruptured intracranial aneurysm, *VEGF* vascular endothelial growth factor, *bFGF* basic fibroblastic growth factor, *OR* odds ratio, *CI* confidence interval

Statistical result for MR analysis is illustrated in Fig. 2 and Additional file 1: Table S4. For main MR analysis employing IVW or Wald ratio methods, no significantly casual effect was observed after Bonferroni adjustment. However, nominal associations with aSAH were observed in VEGF (VEGF→aSAH: OR = 1.15, 95%CI 1.04-1.26, *P* = 0.005, Power = 0.97) and bFGF (bFGF→aSAH: OR = 0.62, 95% CI 0.42–0.92, *P* = 0.02, Power = 0.99). Conversely, no correlation was observed between cytokines and uIA (Fig. 3).

No heterogeneity was identified by Cochran’s Q test for SNPs related to VEGF and bFGF. However, a notable level of heterogeneity was observed in IL-5, IL-6, and IL-17. Directionality was evaluated MR-Steiger pleiotropy and revealed no reverse casual direction. MR-Egger indicated an absence of pleiotropy across all instruments. Similarly, MR-PRESSO provided no evidence of horizontal pleiotropy for tested SNPs except IL-17 (Additional file 1: Table S4). Furthermore, by employing weighted median, MR-Egger, and MR-PRESSO to MR analysis, associations with aSAH at nominal significance were observed in IL-5 (OR = 1.37, 95% CI 1.02–1.86, P_weighted-median_ = 0.04) and IL-13 (OR = 1.12, 95% CI 1.01–1.26, P_weighted-median_ = 0.03). Further sensitivity analysis utilizing *cis*-association did not identify relevant pleiotropy (Additional file 1: Table S8).

In the reverse analysis, a total of 20 SNPs were selected as instrument, with 12 for aSAH and 8 for uIA. No evidence of weak instrument was identified, with *F*-statistics ranging from 20.89 to 73.69 (Additional file 1: Table S2). Analytical results by IVW or Wald ratio are shown in Fig. 2. No significant association was observed after Bonferroni correction, indicating reverse causality was absent for VEGF as well as bFGF. However, there were suggestive effects of uIA (uIA→RANTES: β = 0.122, 95% CI 0.038–0.205, *P* = 0.004; uIA→MIF: β = 0.114, 95% CI 0.032–0.197, *P* = 0.006; uIA→GROα: β = 0.083, 95% CI 0.000–0.165, *P* = 0.05) on circulating biomarkers. Sensitivity analyses did not reveal significantly detrimental results for exposures of interest (Additional file 1: Table S5).

### Potential effect of VEGF and FGF families on IA

In light of the findings presented by primary analysis, which indicated the potential association of VEGF and FGF with risk of IA, external analysis was conducted attempting to identify specific molecular subtypes of VEGF and FGF involved in the associations. Summary statistics from alternative proteomic studies and FinnGen cohort were used [12–14]. SNPs amounting to 488 were obtained at the expanded threshold (P < 5 × 10^-6^) after eliminating the effect of linkage disequilibrium. Examination the availability in outcome data and proxies matching resulted in 453 remaining SNPs as final instrumental variables. These variables represented 7 VEGF and 22 FGF subtypes. Variation explained by individual exposures ranged from 2.17% for FGF-3 to 87.0% for VEGF-R3. *F*-statistics ranged from 20.85 to 1025.5, therefore, provided no evidence for weak instrument (Additional file 1: Table S3).

**Fig. 4:**
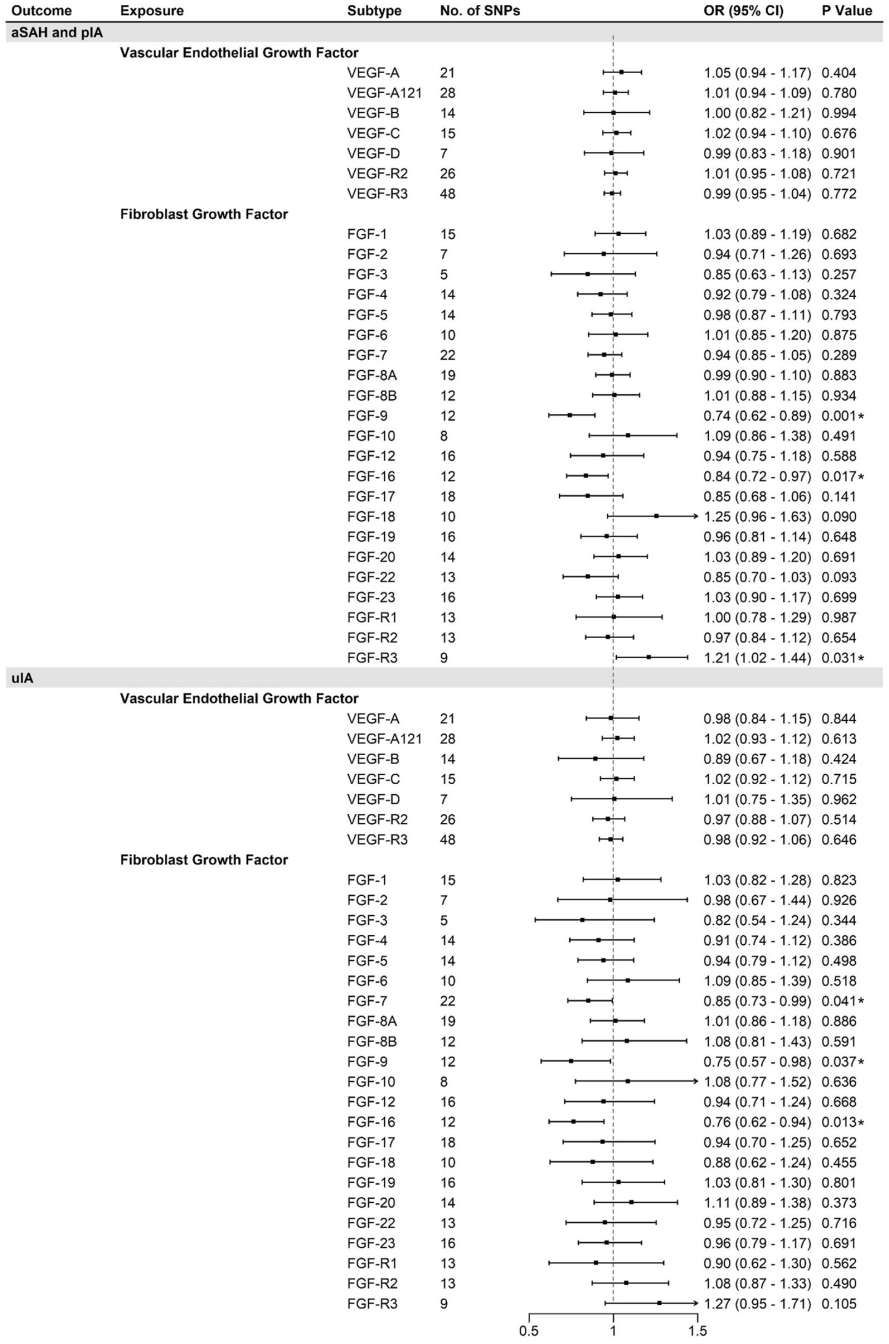
VEGF and FGF families and risk of intracranial aneurysm. *, indicated nominal significance of *P* < 0.05; *aSAH* aneurysmal subarachnoid hemorrhage, *pIA* postoperative intracranial aneurysm, *uIA* unruptured intracranial aneurysm, *VEGF* vascular endothelial growth factor, *FGF* fibroblastic growth factor, *VEGF-R* vascular endothelial growth factor receptor, *FGF-R* fibroblastic growth factor receptor, *OR* odds ratio, *CI* confidence interval

**Fig. 5:**
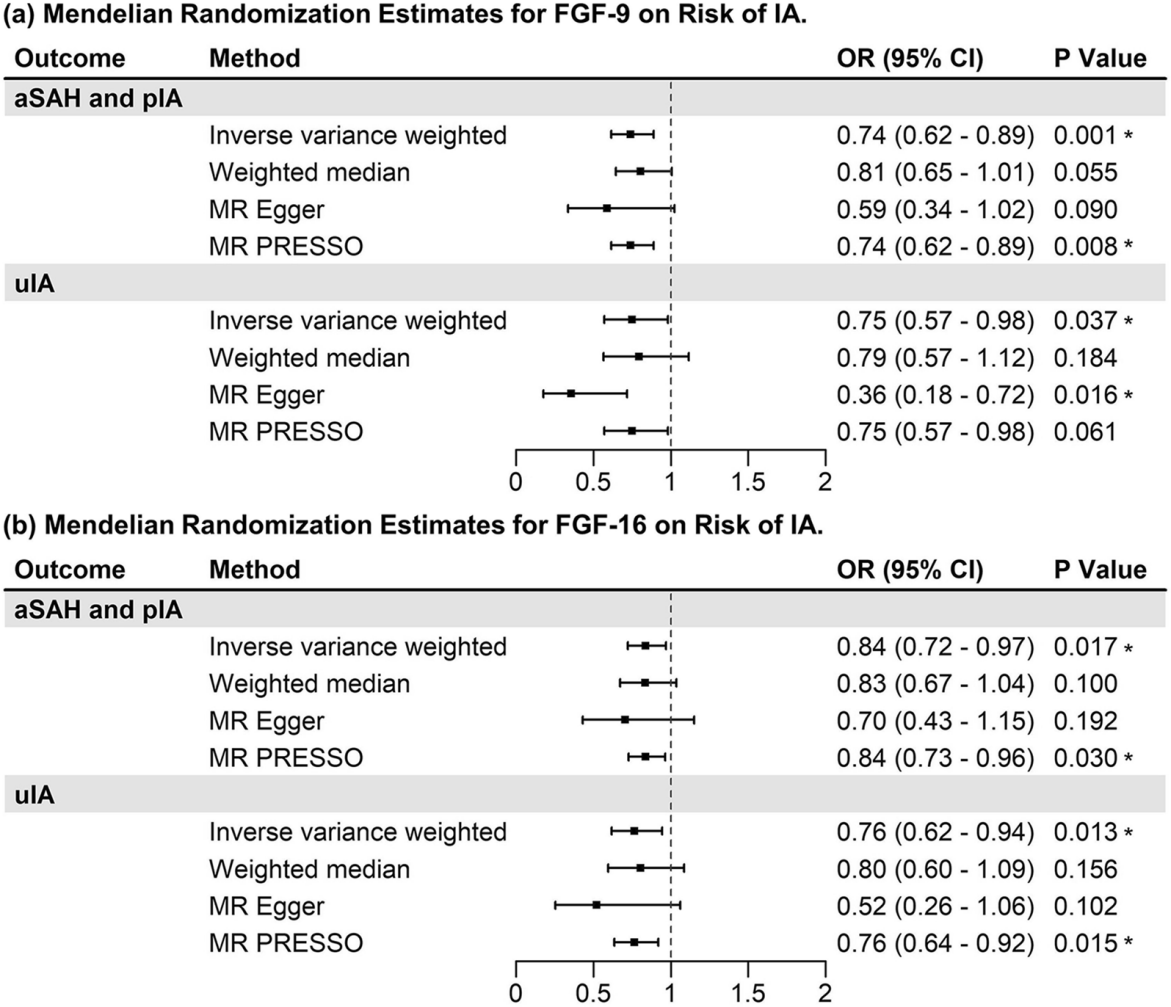
Mendelian randomization estimates utilizing FGF-9 and FGF-16 as exposures. *, indicated nominal significance of *P* < 0.05; *aSAH* aneurysmal subarachnoid hemorrhage, *pIA* postoperative intracranial aneurysm, *uIA* unruptured intracranial aneurysm, *OR* odds ratio, *CI* confidence interval

Overall Mendelian randomization result analyzed by IVW method is shown in Fig. 4 and Additional file 1: Table S6. We did not identify causal relationship between growth factors and risk of IA after Bonferroni correction (*P* < 0.0014). A suggestive inverse association was observed between FGF-9 and aSAH (FGF-9→aSAH: OR = 0.74, 95% CI 0.62–0.89, *P* = 0.001, Power = 1.00). Except for FGF-9, FGF-16 was considered as potential protective factor for aSAH (FGF-16→aSAH: OR = 0.84, 95% CI 0.72–0.97, *P* = 0.017, Power = 0.90), while FGF-R3 as risk factor (FGF-R3→aSAH: OR = 1.21, 95% CI 1.02–1.44, *P* = 0.03, Power = 1.00). Protective effects for uIA were identified in FGF-7, FGF-9, and FGF-16 as illustrated in Fig. 4. Since FGF-9 and FGF-16 both constituted the FGF-9 subfamily, protective function was furthered verified by other MR estimates including weighted median, MR-Egger and MR-PRESSO (Fig. 5). However, the relationships established between VEGF, bFGF and risk of IA in primary analysis were not replicated in the present analysis. Sensitivity analyses did not identify heterogeneity, reverse causality, or horizontal pleiotropy for FGF-7, FGF-9, FGF-16, and FGF-R3. To minimized confounding bias, genotype–phenotype association was screened for the above four FGFs of interest via PhenoScanner, which revealed no associations between SNPs and previously defined confounders. *Cis*-association did not reveal significant pleiotropy for exposures of interest (Additional file 1: Table S9).

Reverse MR analysis employed 5 SNPs for uIA and 13 SNPs for aSAH as instrument. Neither the original nor the proxy SNP was able to identify in 8 out of 29 growth factors, therefore, 21 growth factors were incorporated as final outcome (Additional file 1: Table S3). Utilizing IVW or Wald ratio methods, IA subtypes tended to suggestively associate with FGF-16 (uIA→FGF-16: β = 0.085, 95% CI 0.023–0.146, *P* = 0.007) and FGF-19 level (aSAH→FGF-19: β = − 0.089, 95% CI − 0.166 to − 0.012, *P* 0.02). The association did not reach statistical significance after multiple comparison correction, and sensitivity assessment did not identify adverse results for either FGF-16 or FGF-19 (Additional file 1: Table S7).

## Discussion

In the present study, bidirectional two-sample MR analysis was conducted to systematically evaluate potential casual effect between 41 circulating inflammatory biomarkers and IA. Although no casual effect was observed after Bonferroni adjustment in primary analysis, VEGF and bFGF levels were suggestive to be associated with aSAH, with VEGF acting as risk factors and bFGF as protective factors. Molecular subtypes of VEGF and FGF were further employed for additional analyses. Suggestive protective effect was identified between FGF-9 subfamily and IA, while no correlation was observed between VEGF subfamily and risk of IA in the external group. Moreover, reverse analyses identified suggestive effect of uIA on RANTES, MIF, GRO-alpha, FGF-16, and FGF-19. Sensitivity analyses did not provide evidence for significant heterogeneity or pleiotropy for biomarkers of interest, which reinforced our main findings.

FGFs and corresponding receptors regulate a multitude of biological processes, including organ development, metabolism, and pathological processes. Six subfamilies have been identified in FGF family, among which, the FGF-9 subfamily comprises FGF-9, FGF-16, and FGF-20 [31]. Few observational studies had been conducted to investigate the relationship between FGFs and IA, most of which focused on FGF-2 (also known as bFGF). It was reported that higher expression level of FGF-2 was associated with IA and might contribute to vascular remodeling [32], while contradictory finding revealed that the expression pattern of FGF-2 did not differ in normal arterial, ruptured aneurysm, or unruptured aneurysm wall [33, 34]. Evidence provided by in vitro and in vivo experimental studies showed that cellular proliferation, tissue fibrosis, and vascular wall thickness were enhanced by FGF-2 in aneurysm models [35, 36]. Therapeutic potential was observed in exogeneous FGF-2 that it could inhibit the dynamic growth of aneurysm [37]. It was consistent with our findings in primary analysis, which identified FGF-2 as a suggestive protective factor for IA. However, statistical significance for FGF-2 diminished when we expanded the exposures to the entire FGF family in external analysis, where nominal inverse associations with risk of IA were further observed in FGF-9 and FGF-16, namely the FGF-9 subfamily. The FGF-9 subfamily was currently identified as regulators for the development of skeletal, respiratory, urinary, and cardiac system [31]. Genetic deficiency of FGF-9 had been observed in several pathological conditions, including abnormal bone repairment, idiopathic pulmonary fibrosis, myocardial infarction, and tumorigenesis [31, 38]. Nevertheless, the association of FGF-9 and IA has yet been established, since neither epidemiological nor experimental studies had been performed to directly investigate the effect of FGF-9 on IA. We noticed suggestive protective effects of FGF-9 subfamily against both aSAH and uIA in the present MR study, however, the underlying molecular mechanisms remained elusive and controversial. On one hand, FGF-9 was capable of inducing proinflammatory environment and thereby exacerbated neuropathological process, which was achieved by the induction of CCL2, CCL7, and inhibitors of MMP-sensitive proteases [39]. On the other hand, FGF-9 exhibited the capability to recruit vascular smooth muscle cell and produce vasoresponsive microvessels, which was critical for tissue repairmen [40, 41]. The conflicting findings of previous studies were restricted by limited sample sizes and the examination of different or specific tissue types. Consequently, conducting future research specifically targeting the aneurysmal wall might be valuable for understanding the precise role of FGF-9 in IA. Additionally, we observed suggestive effect of IA on FGF-16 and FGF-19 in reverse analysis. While FGF-16 may share the same molecular mechanism with FGF-9 in a paracrine manner, FGF-19 was considered as an endocrinal regulator in energy metabolism. Expression of FGF-19 was concentrated in human ileum and gallbladder epithelial cells, responsible for bile acid synthesis, glycogen synthesis, and lipogenesis [31]. However, no observational or experimental studies had been conducted to evaluate the association between IA and FGF-19. The lack of evidence posed challenges in confirming the precise role of FGF-19 in the development of IA.

The primary subtype of VEGF family, VEGF-A, is essential for angiogenesis and associated pathological process [42]. The associations between VEGF-A level and IA were investigated by previous observational studies. Plasma VEGF concentrations were found to be increased uIA patients compared with healthy controls, but statistical significance was restricted to male participants [43]. Controversial result was carried out by another study which did not identify significant difference of VEGF in plasma but rather in cerebrospinal fluid [44]. Experimental studies further indicated the role of VEGF-A in aneurysmal growth and rupture [45, 46]. MR result presented in our primary analysis was partially consistent with previous findings, however, we could not replicate the outcome at suggestive significance in external analysis. The hypoxia-inducible factor-1 alpha (HIF-1α) signaling pathway may explain the potential molecular mechanism for VEGF-A involving in IA, especially aSAH. Cell apoptosis, blood–brain barrier disruption, and brain edema after aSAH was triggered by HIF-1α along with the upregulation of VEGF, while inhibition of HIF-1α, VEGF, and MMP-9 showed less neurological lesion in aSAH models [46, 47].

In addition, the present MR study did not identify other significant inflammatory biomarkers with the given datasets in forward analyses. Although IL-5 and IL-13 reached nominal significance applying the weighted median method, we did not consider them as candidate factors due to negative outcome utilizing IVW method. Conversely, our result in reverse MR indicated that multiple inflammatory biomarkers, including IL-10, RANTES, MIF, and GRO-alpha, were suggestively affected by intracranial aneurysm and its subtypes. Evidence derived from conventional studies was limited and controversial but could still provide insights for the role of proinflammatory factors. Our finding was partially consistent with a previous study which observed higher plasma levels of RANTES, MCP-1, MIG, IP-10, eotaxin, IL-8 and IL-17 were found in the lumen of human cerebral aneurysms [48]. It was further confirmed by another research which found elevated serum and cerebrospinal fluid levels of RANTES after aSAH and indicated RANTES was independently associated with clinical outcome [49]. Result of the present study was consistent with previous research which observed significantly higher cerebrospinal fluid level of GRO-alpha in uIA patients [50]. Our result was supported by a previous MR study which showed sIL6R and CRP levels were not associated with IA [8]. Our finding was partially contradictory to a meta-analysis, which found no evidence of IL-1α, IL-1β, IL-6, and IL-12β against IA, but identified significant association of TNF-α polymorphism with IA [51]. Still, current epidemiological and experimental evidence for the role of inflammatory biomarkers in the formation and progression of IA remained insufficient, and thereby required future research to unveil the mystery.

To the best of our knowledge, this is the first two-sample MR study to systematically evaluate the effect of circulating inflammatory cytokines and growth factors on IA. By utilizing bidirectional MR analysis, we managed to minimize potential bias from reverse causation. However, several limitations existed. First, false-positive variants might have been included as we used a higher selection threshold of *P* < 5 × 10^–6^. However, a more stringent threshold (*P* < 5 × 10^–8^) results in less available instrumental variables, leading to less statistical power and insufficient sensitivity analyses. Still, multiple prior studies had employed the relaxed threshold to investigate the relationship between cytokines and diseases [52, 53]. Second, although we performed pleiotropy test and utilized PhenoScanner, the potential bias from confounders could not be statistically ruled out. Third, the classification of aneurysmal hemorrhage group in FinnGen dataset remained ambiguous. Both aSAH and pIA participants were included in one single group, therefore, we could not determine whether cytokines correlations were interrupted by surgical interventions. Fourth, since the distribution of genetic polymorphisms may differ among populations, we collected summary GWAS statistics restricted to European descent. The generalizability of result to other populations remained undetermined. Finally, we identified inverse suggestive associations of FGF-9 subfamily with risk of IA in external analysis. However, current statistical methods could not identify the exact affected stage, namely, whether formation or progression of IA was associated with the significant cytokines. Further prospective cohort studies could provide insight for the temporal dynamics of cytokines in conjunction with IA.

## Conclusions

In summary, the current study employed bidirectional two-sample MR analysis to systematically assess causality of inflammatory biomarkers, especially FGFs and VEGFs, on risk of IA subtypes. While no causal effect was identified after Bonferroni correction, we suggested that FGF-9 and FGF-16 may be protective factors for aSAH. Although nominal effect of bFGF and VEGF were observed in primary analysis, statistical significance failed to replicate in external analysis. Future studies are required to determine the temporal dynamics of cytokines in conjunction with IA.

### Supplementary Information

**Additional file 1: Table S1**. Description of genome-wide association studies and datasets included in the present study. **Table S2**. Statistical characteristics of instrumental variables included in the primary analysis. **Table S3**. Statistical characteristics of instrumental variables included in the targeted analysis. **Table S4**. Mendelian randomization estimates and sensitivity analyses for the effect of inflammatory biomarkers on intracranial aneurysm. **Table S5**. Mendelian randomization estimates and sensitivity analyses for the effect of intracranial aneurysm on inflammatory biomarkers. **Table S6**. Mendelian randomization estimates and sensitivity analyses for the effect of vascular endothelial growth factors and fibroblast growth factors on intracranial aneurysm. **Table S7**. Mendelian randomization estimates and sensitivity analyses for the effect of intracranial aneurysm on vascular endothelial growth factors and fibroblast growth factors. **Table S8**. Mendelian randomization estimates and sensitivity analyses for the effect of inflammatory biomarkers on intracranial aneurysm utilizing *cis*-association. **Table S9**. Mendelian randomization estimates and sensitivity analyses for the effect of vascular endothelial growth factors and fibroblast growth factors on intracranial aneurysm utilizing *cis*-association.

**Additional file 2: **STROBE-MR checklist of recommended items to address in reports of Mendelian randomization studies.

## Data Availability

Summary statistics utilized in the present study were extracted from published articles and publicly archived datasets, with hyperlinks listed in Additional file 1: Table S1.

## Abbreviations

aSAH: Aneurysmal subarachnoid hemorrhage
bFGF: Fibroblast growth factor basic
CI: Confidence interval
COX-2: Cyclooxygenase‑2
CRP: C-reactive protein
CTACK: Cutaneous T cell-attracting chemokine
FGF: Fibroblast growth factor
G-CSF: Granulocyte colony-stimulating factor
GRO-alpha: Growth-regulated protein alpha
GWAS: Genome-wide association study
HGF: Hepatocyte growth factor
HIF: Hypoxia-inducible factor
IA: Intracranial aneurysm
IL: Interleukin
IP: Interferon-gamma-inducible protein
IV: Instrumental variables
IVW: Inverse variance weighted
MCP: Monocyte chemoattractant protein
M-CSF: Monocyte colony-stimulating factor
MIF: Macrophage migration inhibitory factor
Mig: Monokine induced by interferon-gamma
MIP: Macrophage inflammatory protein
MR: Mendelian randomization
MR-PRESSO: Mendelian randomization pleiotropy residual sum and outlier
NGF: Nerve growth factor
OR: Odds ratio
PDGF-BB: Platelet-derived growth factor-BB
pIA: Postoperative intracranial aneurysm
RANTES: C–C chemokine ligand 5
SCF: Stem cell factor
SCGF: Stem cell growth factor
SD: Standard deviation
SDF: Stromal-derived factor
SNP: Single nucleotide polymorphism
TNF: Tumor necrosis factor
TRAIL: TNF-related apoptosis-inducing ligand
uIA: Unruptured intracranial aneurysm
VEGF: Vascular endothelial growth factor
